# Computer-assisted image processing to detect spores from the fungus *Pandora neoaphidis*

**DOI:** 10.1016/j.mex.2016.03.011

**Published:** 2016-03-17

**Authors:** Reinert Korsnes, Karin Westrum, Erling Fløistad, Ingeborg Klingen

**Affiliations:** a Norwegian Defense Research Establishment (FFI), Box 25, N-2027 Kjeller, Norway; b Norwegian Institute of Bioeconomy Research (NIBIO), Biotechnology and Plant Health Division, P.O. Box 115, NO-1431 Ås, Norway

**Keywords:** Biological control, Computerised spore detection, Pathogenic fungus

## Abstract

This contribution demonstrates an example of experimental automatic image analysis to detect spores prepared on microscope slides derived from trapping. The application is to monitor aerial spore counts of the entomopathogenic fungus *Pandora neoaphidis* which may serve as a biological control agent for aphids. Automatic detection of such spores can therefore play a role in plant protection. The present approach for such detection is a modification of traditional manual microscopy of prepared slides, where autonomous image recording precedes computerised image analysis. The purpose of the present image analysis is to support human visual inspection of imagery data – not to replace it. The workflow has three components:

• Preparation of slides for microscopy.
• Image recording.
• Computerised image processing where the initial part is, as usual, segmentation depending on the actual data product. Then comes identification of blobs, calculation of principal axes of blobs, symmetry operations and projection on a three parameter egg shape space.

## Method details

**Fig. 1:**
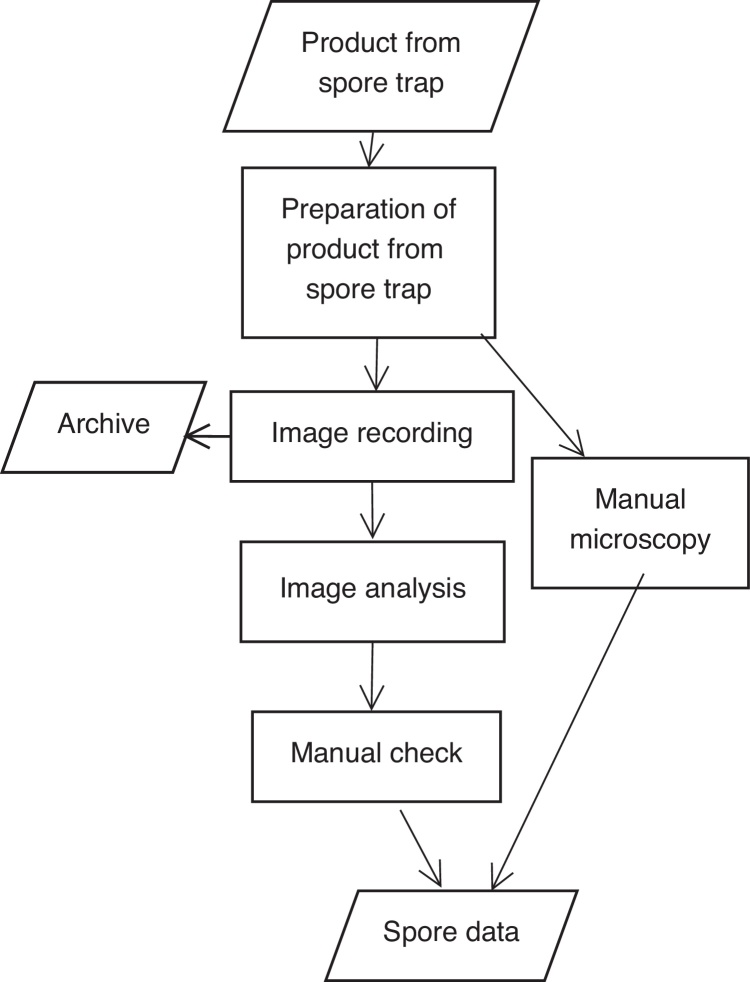
Workflow for sensing spore counts in air.

Monitoring spore counts in air using spore trap samplers traditionally includes laborious counting of spores under microscope before subsequent statistical treatment. The present contribution reports an attempt to apply computerised image analysis to ease this part of this workflow without modifying instrumentation and slide preparation (Fig. 1). The approach may, however, allow for different requirements on slide preparation. Automatic microscopy will normally include posterior manual inspection. So visual properties of imagery products are still relevant.

The image analysis below applies to an investigation on how an entomopathogenic fungus may serve as a biological control agent for aphids. Many types of fungi are important entomopathogens suppressing insect populations [8]. The study fungus here is *Pandora neoaphidis* (Syn. *Erynia neoaphidis*). It is within the phylum *Entomophtoromycota*, class *Entomophthoromycetes* and order *Entomophthorales* [12]. The fungus is an important pathogen on aphids in temperate agroecosystems where it can cause epizootics and control their population on local scale [6], [7], [8], [9]. Monitoring and predicting spores from *P. neoaphidis* is therefore relevant to use in pest management decision support systems.

Conservation biological control with fungal natural enemies of pest insects and mites is possible. One way of doing this is to reduce pesticide use in critical periods to avoid harm to these natural enemies. Predictions of potential suppression of pest insects and mite populations by fungal pathogens are based on monitoring of the natural fungal infection level in a pest population. Results from this monitoring may then be used in a prediction model for the epidemic development and hence control of a pest population by fungal natural enemies. These prediction models might then be used in decision support systems (DSS).

Attempts have been made in USA to prevent pesticide treatment of mite populations on soya crops when they are suppressed by fungal epizootics [15]. Similar approaches have been undertaken for the related insect pathogenic fungus *Neozygites fresenii*, infecting cotton aphid (*Aphis gossypii*). Growers withhold insecticide application for aphids when they expect *N. fresenii* epizootics to control the cotton aphid [10]. This rule of engagement traditionally depends on laborious collection and squash mounting of aphids to collect data on fungal propagules in/on the aphid. Utilisation of spore traps and computer-aided processing could ease the situation assessment.

The life cycle of *P. neoaphidis* starts with a conidia (spore) fixing to the cuticle of an aphid where it germinates and penetrates into the insect. It then gradually fills the aphid with protoplasts and produces hyphal bodies close to and after the dead of the host [4]. Spore bearing structures (conidiophores) subsequently break out through the surface of the cadaver where it releases primary conidia (sporulation). This sporulation requires a microclimatic relative humidity (RH) above 93% [22]. *P. neoaphidis* may also form resting spores *in vivo* in aphids and activate later under special conditions [19].

A conidium can produce secondary conidia if it does not adhere to a suitable surface (another aphid-cuticle). Both primary and secondary conidia of *P. neoaphidis* are infective [5]. The shape of a primary conidium is clavate and obovoid with a rounded basal papilla and it has a length of 15–40 μm and a width of 9–16 μm [11]. Secondary conidia have the length of 16–25 μm and a width of 7–15 μm and can be of the same shape or more rounded than the primary conidia [14].

Monitoring spore counts in air using spore trap samplers traditionally includes laborious manual counting of spores under microscope before subsequent statistical treatment. The accuracy of the results may depend on the researcher's experience. Several authors have therefore tried to develop automatic identification of spores in images. Benyon et al. [2] made such an attempt including extraction of 7 basic shift-rotation invariant features: length, width, width-length ratio, area, form factor, perimeter and roundness. They also applied several more complex features such as area of convex hull.

Bonner et al. [3] approached computerised measurement of production of conidia from the aphid pathogenic fungus *Erynia neoaphidis*. They focused on data preparation to simplify the computerised part of the workflow.

Ranzato et al. [17] summarised previous work on recognition of biological particles in microscopic images. They approached the problem by a preliminary search for interesting locations in images followed by estimation of parameters of brightness at different scales. A mapping into a feature space provided rotation and translation invariant regional features subject to classification to distinguish between different types of particles.

Complex data preparation/recording and use of domain knowledge may help to simplify image processing to identify spores in images. The present contribution attempts to aid identification of spores in images of microscope slides originally meant for manual processing. It may be regarded as a possible low cost, simple and intuitive extension of established manual and visual skill-based procedures.

Molecular methods to detect airborne spores are developing [16], [18]. This approach requires design and development of DNA primers, DNA extraction techniques and PCR-based methods suitable to detect, clone and sequence spores in question. Such molecular detection methods exist for most of the entomopathogenic hypocrealean fungi, but there are only few for entomopathogenic fungi in the Entomophthoramycota.

## Materials and methods

### Pandora neoaphidis isolate

The present experiments include use of a *P. neoaphidis* isolate NCRI 393/13 obtained from its natural host the English grain aphid (*Sitobion avenae*) on wheat (*Triticum aestivum*) at Horten (WGS84: N59°26.083′, E10°24.191′), Norway, 8 August 2013. The *P. neoaphidis* isolate was cultured on Saboraud Dextrose Milk Yolk Agar (SDAMY) in sterile Petri dishes (diameter 5 cm) sealed with Parafilm, and transferred onto new Petri dishes regularly to ensure maintenance of the culture by cutting three circular pieces (5 mm) from the edges of the fungal mat and transferring these to new Petri dishes with SDAMY. The Petri dishes were kept in dark plastic boxes lined with wet filter paper to ensure high humidity and placed at 18 °C and 65% RH.

### Experimental setup

*Myzus persicae* was used in this controlled spore discharge experiment and 20 adult female *M. persicae* were placed on a 25 mm paprika leaf disc in a 55 mm petri dish with 1.5% water agar in darkness at 18 °C and 70% RH for 24 h prior to the inoculation for the aphids to settle before exposure to the fungal isolate. After 24 h, aphids were exposed to *P. neoaphidis* by placing a Petri dish with a sporulating culture of the pathogen on SDAMY over the petri dish with aphids. A fine plastic gauze with mesh size 1 mm × 0.5 mm was put in between the aphid dish and the lid, to avoid the aphids getting in direct contact with the inoculum [1], [20]. The dishes with aphids and fungal cultures were kept in dark plastic boxes with wet filter paper for high humidity, and left to sporulate for 5 h at 15 °C and 70% RH. The fungal cultures were removed after 5 h and the aphids transferred onto two plants in a small wind tunnel. The plants were placed 10 cm apart in the centre of the tunnel. Two leaf discs, each with 20 *P. neoaphidis*-inoculated *M. persicae*, were placed on each of the two plants in the tunnel providing 40 *P. neoaphidis* inoculated *M. persicae* on each plant.

The wind tunnel consisted of light transmitting plexiglass that was equipped with a rotating spore trap cylinder that was designed as described by Suthaparan et al. [21]. Close to the opening of the tunell, a spore trap of 1.5 L plastic bottle with sticky tape was placed on a 24 h rotating timer. The timer ensures one complete rotation of plastic bottle for 24 h. Each spore trap consisted of Melinex microscope tape, 345.0 mm, fitted around 1.5 L bottles. A solution of 9.0 g clear vaseline, 1.0 g fluid parafin and 100.0 ml of toluene was heated in warm water and added onto the tape with a paintbrush. The toluene solution ensured that the spores would stick to the tape and be conserved until counting. Spore trap was changed daily at 10:00 in all six tunnels during the experiments. The tape on each spore trap was removed, placed in plastic boxes and stored in a fridge at 5 °C until counting of spores.

To count the spores from *P. neoaphidis*-killed *M. persicae* cadavers on plants, the Melinex tape from the spore trap was cut into six 4.9 cm pieces representing 4 h on each piece of the 24 h cycle. Each piece of tape was held in place by two drop of glycerol on the microscope slides. Two drops of a staining solution consisting of 0.075% cotton blue in 50% lactic acid were added on top of each piece of tape, and a cover slip (50 mm × 23 mm) was then placed on top. The spores were manually/visually counted in vertical transects of 2.0 mm intervals (representing 10 min) in each reading under a phase contrast microscope (100×) resulting in a total of 144 transect readings per 24 h.

## Microscopy imagery data

**Fig. 2:**
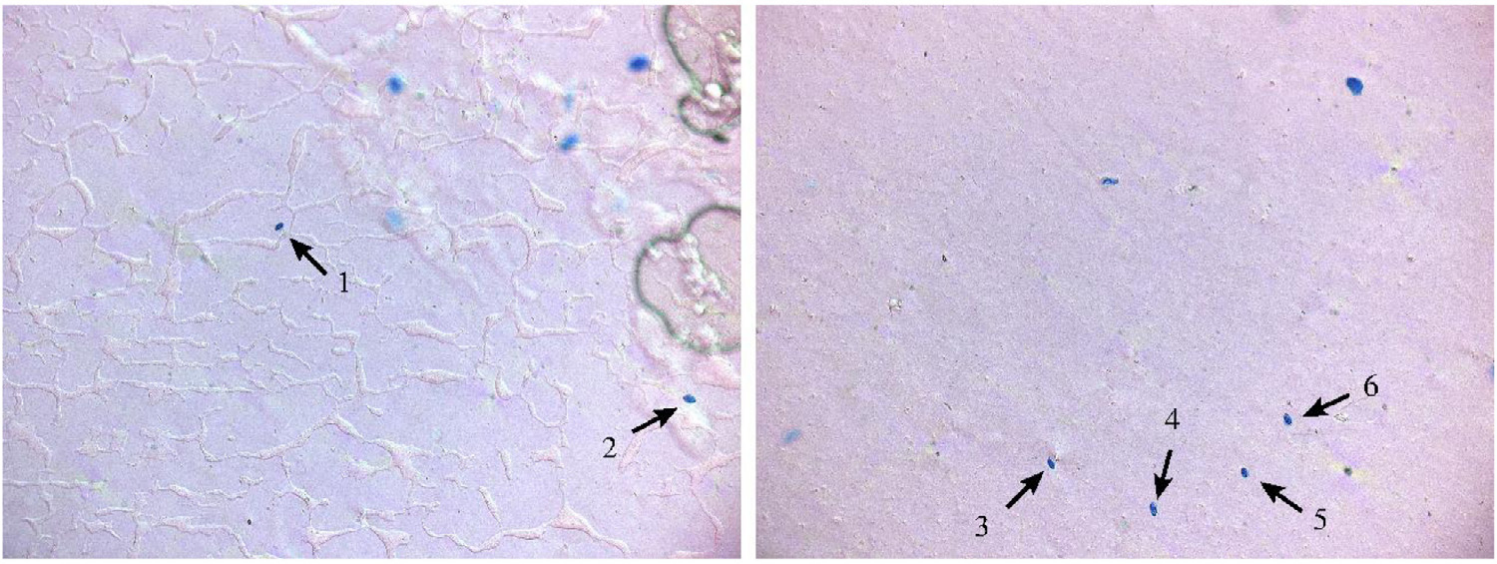
Two examples of the present type of images showing 2 and 4 spores respectively (from left to right). Note that the spore detection algorithm has to distinguish spores from similar objects (blue dots). (For interpretation of the references to colour in this figure legend, the reader is referred to the web version of the article.)

Microscope slides containing spores trapped during 4 h of collection were batch photographed using a microscope of type Leica DM 6000 B, fitted with a CTR 6000 control unit, and a DFC 425 camera. Images were captured at 10× magnification (Leica HC PL 10×/0,40) giving a pixel dimension of 0.5063 μm. Focal plane was determined from focusing at 10 randomly selected spores, at different areas of the slide, and selecting the mean focal plane for batch photo. The Leica LAS-Multistep-module was used to capture a grid of 760 images, covering the entire slide. Initially stored TIF images files were converted to jpeg format using Adobe Photoshop Lightroom 5.7. Fig. 2 shows two examples of images resulting from the present procedure. A total of 765 images were produced to test the present approach for image processing. Approximately thirty percent of these images contained findings of possible spores for manual check.

## Image analysis

### Data product dependency

A digital image of the surface of a real object can typically be looked at (in mathematical terms) as a measure of distributed energy emission from the surface and restricted to a frequency band. A picture element (pixel) represents a part of the surface and its (pixel) value then represents energy emission from that part (at least approximately). However, the physical dimension (unit) of a pixel value is normally not well defined (i.e. the data is not physically calibrated). Assume the pixel *P*_*i*_ has numerical value *x*_*i*_ (*i* = 1, 2). If *x*_1_ < *x*_2_ then one may believe that *x*_1_ + *x*_2_ represents radiation from the area *P*_1_ ∪ *P*_2_. However, this is not the case for non-calibrated data, and the result of processing such data may therefore depend on preparation and scanning of slides.

Estimates of the gradient of pixel values in an image, for example, includes arithmetic operations on pixel values. If these numerical values do scale properly with radiation, then the gradient of pixel values may not line up with the gradient of radiation from the physical object. However, images often exhibit structures which an algorithm may identify. The algorithm and its parameters will in this case depend on the data product. Sections “Spore colour space” and “Image segmentation” below is an attempt to isolate the data preparation dependent part in the processing facilitation design of a generic geometric approach.

### Spore colour space

**Fig. 3:**
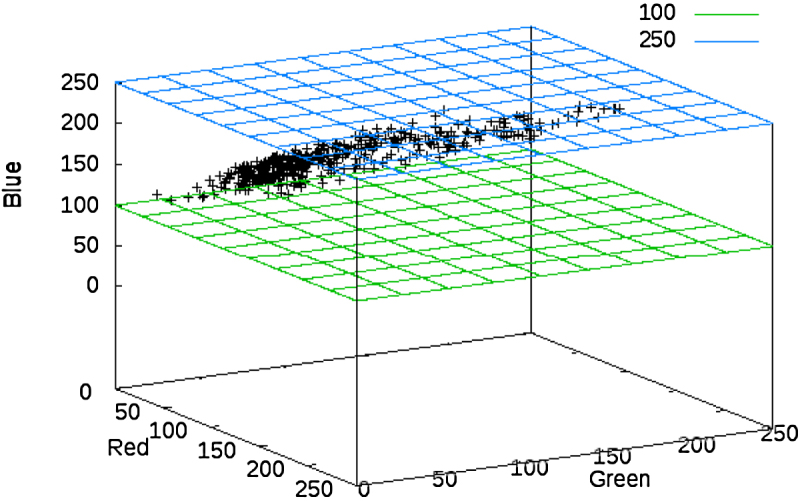
Example of colour composition of (RGB) values of pixels interior spores in present images. The value of the blue component ranges between 100 and 250. Small values correspond to small values of red and green (and *vice versa*). (For interpretation of the references to colour in this figure legend, the reader is referred to the web version of the article.)

Fig. 3 gives a typical example of the red, green and blue (RGB) values of pixels included in spores on the actual images. A three-dimensional vector $r=(R,G,B)\in {\mathbb{R}}^{3}$ in this case represents the red (R), green (G) and blue (B) component of a pixel value in the numerical range 0–255 (here noted as a RGB-vector). The figure shows that the different colours strongly correlate. The RGB-vector values for a spore form a linear shaped structure embedded in the three-dimensional space ${\mathbb{R}}^{3}$.

**Fig. 4:**
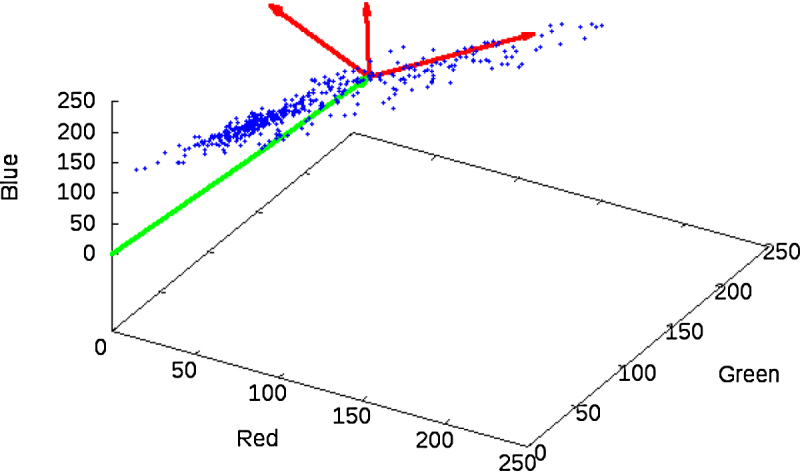
Example of colour composition of (RGB) values of pixels interior spores in present images. (For interpretation of the references to colour in this figure legend, the reader is referred to the web version of the article.)

A standard principal component analysis reflects the above observation of correlation between colour components for a spore. Let ***A*** be the correlation (positive definite) matrix for the RGB vector. Let *λ*_1_ ≥ *λ*_2_ ≥ *λ*_3_ be the ordered set of eigenvalues of ***A*** with corresponding (orthonormal) eigenvectors $v_{1},v_{2},v_{3}$. Fig. 4 illustrates these eigenvectors (red) centered at the mean point $\bar{r}$ (green). The red arrow along the linear shape illustrates the eigenvector $v_{1}$ corresponding to the largest eigenvalue *λ*_1_. It seems to reflect the significant part of the variation of the RGB vector. The mean square deviation of the RGB vector ***r*** from its mean value is Var[***r***] = *λ*_1_ + *λ*_2_ + *λ*_3_, where the first term dominates. A possible measure of the likelihood for an RGB vector ***r*** = (*r*_1_, *r*_2_, *r*_3_) to be from a spore, is

(1) $$s(r)=\sum_{i}\frac{r_{i}^{2}}{\lambda_{i}}$$

A pixel is here classified as being within the colour distribution of a spore if

(2) $$s(r)<P_{1}$$

where *P*_1_ depends on the data product/treatment. This measure can facilitate image segmentation and control of spore identification.

### Image segmentation

#### Gradient methods

Eq. (1) above defines a scalar field $s:{\mathbb{R}}^{2}\to \mathbb{R}$. Assume linearisation of the field (*s*) around the position $\bar{r}\in {\mathbb{R}}^{2}$:

(3) $$s(r)=\nabla s(\bar{r})\cdot (r-\bar{r})+s(\bar{r})+\epsilon$$

where ∇*s* is a vector (gradient of *s*) and *ϵ* represents the error.

The following procedure gives a least square estimate of the gradient ∇*s*. Let the vectors ***r_1_***, ***r_2_***, …, ***r_n_*** represent the positions of *n* pixels surrounding a point $\bar{r}$ in an image. The vector $\Delta r_{i}=r-\bar{r}$ in this case represents the local position (displacement) relative to the mean vector $\bar{r}$. The actual Gram matrix ***G*** becomes a sum of outer products:

(4) $$G=\sum_{i}\Delta r_{i}⊗\Delta r_{i}$$

and measurement vector:

(5) $$m=\sum_{i}s(r_{i})\cdot \Delta r_{i}$$

This gives the following estimate of the gradient of the scalar field $s:{\mathbb{R}}^{2}\to \mathbb{R}$:

(6) $$\tilde{\nabla s}=G^{-1}m$$

A smooth linear border between regions of much different values of *s* gives neighbourhoods of relatively large parallel gradients normal to it. Averaging of gradient values locally along these borders therefore tend to enhance such smooth borders and hence borders of images of spores which have smooth surfaces.

**Fig. 5:**
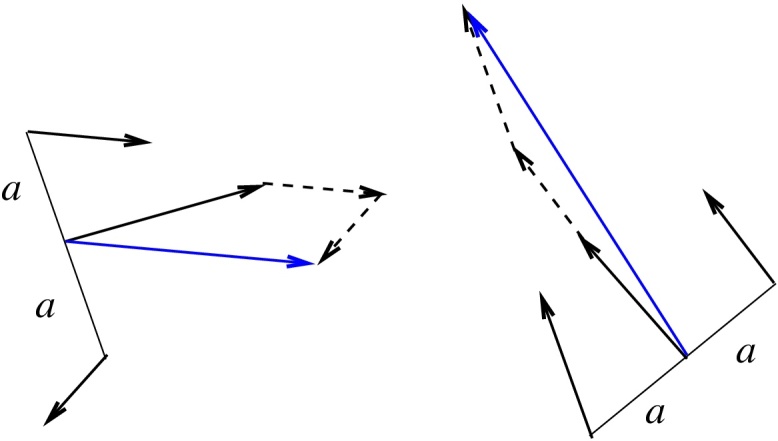
Illustration of sensitivity of vector averages to local directional tendencies along linear features.

Assume the gradient estimate ***g*** = ∇ *s*(***r***) at position ***r*** in an image as above. The normalised vector ***n*** = ***g***/|***g***| denotes its direction (a vector of unit length). The vector ***p*** = *a****Rn***, where ***R*** is the 90° rotation, denotes the position *a* units from the position ***r*** in the direction normal to the vector ***n***. The similar point in the opposite direction is given by −***p***. Fig. 5 illustrates that the sum (or average) of the gradients at the position ***r***, ***r*** + ***p*** and ***r*** − ***p*** is sensitive to tendencies in direction:

(7) $$\bar{g}=\frac{1}{3}[\tilde{\nabla s}(r)+\tilde{\nabla s}(r+p)+\tilde{\nabla s}(r-p)]$$

The variable *a* is here considered to be a learning variable (*P*_2_). The average gradient contributes to reduce noise for the present application of image analysis.

**Fig. 6:**
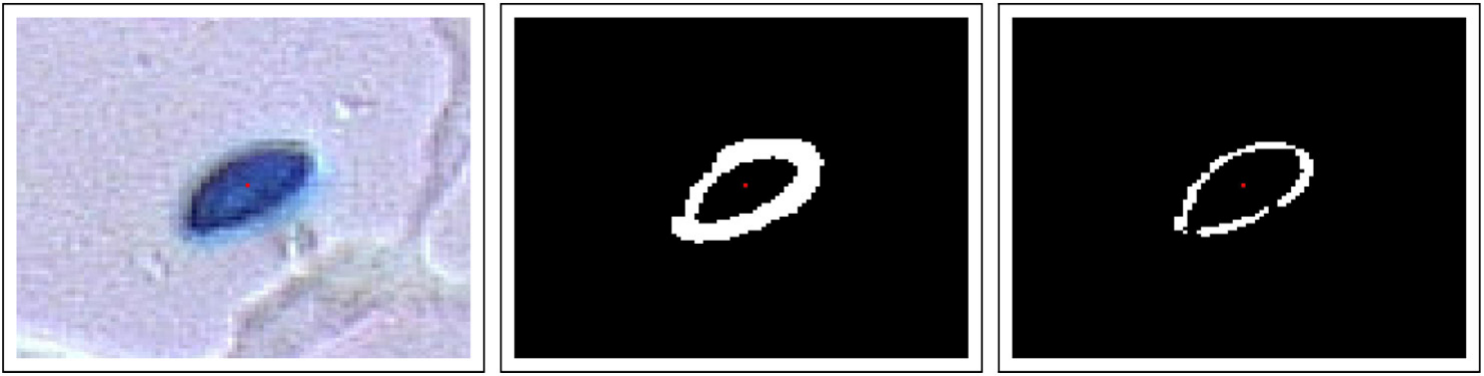
Segmentation of image based on average gradient (cf Eq. (7)). Left image: subset of left image of Fig. 2 of size 100 × 100 pixels (approximately 50 μm across). Middle image: pixels with absolute gradient values $\bar{g}=\bar{g}>P_{3}$ are white (others are black). Right image: pixels with values of $\bar{g}$ above the 25 percent quantile for the neighbourhood pixels condition on $\bar{g}>P_{3}$. A neighbourhood of a pixel is here defined as a square region of 7 × 7 pixels centred at the pixel.

The right image of Fig. 6 illustrates a further refinement of the segmentation based on the average gradient providing less sensitivity to the threshold value *P*_3_. The white pixels are in this case pixels with values above *P*_3_ but in addition they are “extreme pixels” in the way that they have few neighbouring pixels with higher values. This condition provides exclusion of sloping areas of the scaler field $s:{\mathbb{R}}^{2}\to \mathbb{R}$ (cf Eq. (1)), and it contributes to make the pixel classification less sensitive to the threshold *P*_3_.

#### Identification of blobs

**Fig. 7:**
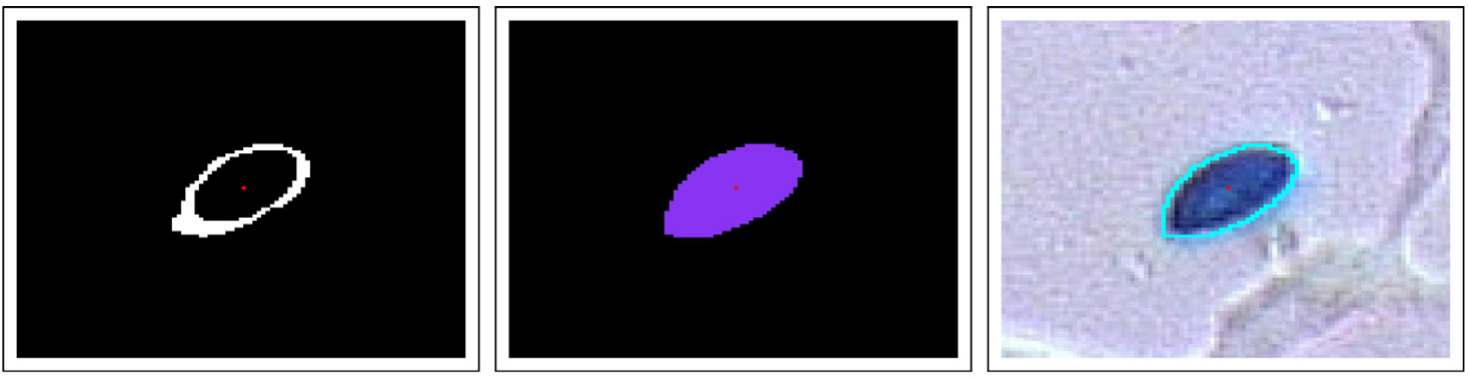
Production of blob by further processing of image of Fig. 6. Left: morphological closing where disc of radius 3 μm is the structuring element. Middle: result from identification of convex hull and subsequent morphological opening where a disc of radius 2 μm is the structuring element. Right: border of blob superimposed on original image.

The gradient method above facilitates identification of blobs in an image [13]. Morphological openings and closing in addition of production of convex hull of connected regions can provide input to further shape analysis. Fig. 7 illustrates the effect of morphological closing of segmented images (as in Fig. 6). Such identification of separate blobs facilitate effective representations in a computer program (in this case Containers in Ada 2012^1^).

#### Axis of blobs

Estimates of the middle axis along a spore is useful for check of symmetry and to initiate form fitting to the perimeter of spores. Section “Fitting egg shape to spore perimeter” illustrates such form fitting where the actual shape is egg formed.

Assume the vectors ***r***_1_, ***r***_2_, …, ***r***_*n*_ denote the positions of pixels of a blob in an image (the middle image of Fig. 7 illustrates such a blob). Let $\bar{r}=1/n\cdot \sum_{i}r_{i}$ be their centre point and ***n*** a unit vector (of length 1). Without loss of generality, assume for simplicity that $\bar{r}$ is the zero vector. The length of the cross product vector ***n*** × ***r***_*i*_ is the distance from the position ***r***_*i*_ and normal to the line defined by ***n***. Hence minimisation of the following sum of cross products defines an axis for the blob:

(8) $$S_{↓}=\sum_{i}|n\times r_{i}{|}^{2}$$

Note that the terms of the sum in Eq. (8) process the equality |***n*** × ***r***_*i*_|^2^ = |***r***_*i*_|^2^ − (***n*** · ***r***_*i*_)^2^. Hence a value of the ***n*** which *minimises S*_↓_, *maximises*

(9) $$S_{\to }=\sum_{i}{\left(n\cdot r_{i}\right)}^{2}$$

### The egg shape space

Keller [14] and Humber [12] described the form as clavate and obovoid with a rounded basal papilla. However, morphological opening (with a disc as structuring element) of the 2-dimensional projection (profile) of spores tend to resemble the form of eggs or ovals. There are several proposals for simple formulas for egg shapes and ovals.^2^ The present work elaborate parameterisation of an “egg shape” based on two simple transformations of the unit disc (*D*):

(10) $$F_{c}:(x,y)\to (x,\;g(x)\cdot y)$$

and

(11) $$G_{a,b}:(x,y)\to (a\cdot x,\;b\cdot y)$$

where *g*(*x*) = *c* · *x* + 1. The three parameters *a*, *b* and *c* (real numbers) in this case define an egg shape *S* as the image of the unit disc *D*:

(12) $$S=\{G_{a,b}\circ F_{c}(r)|r\in D\}$$

**Fig. 8:**
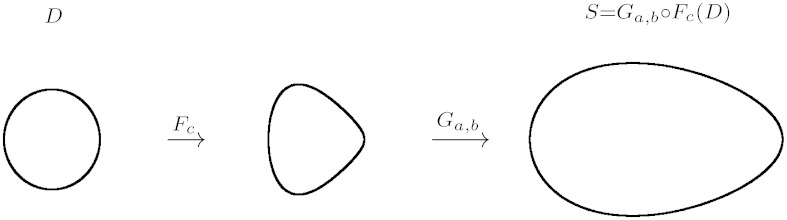
A three-parameter egg shape as an image of the unit disc by composite function *F*_*c*_ ∘ *G*_*a*,*b*_ (cf Eqs. (10), (11), (12)). In this case *a* = 2.5, *b* = 1.5 and *c* =−0.5.

Fig. 8 illustrates this composite mapping.

#### Fitting egg shape to spore perimeter

**Fig. 9:**
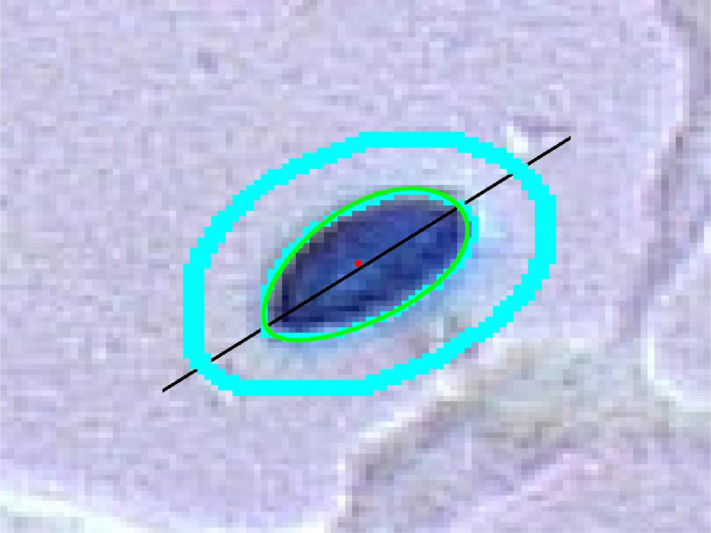
Fitting egg shape to spore perimeter.

Fig. 9 gives an example of a egg shape fit to the perimeter of a blob in an image. The goodness of fit in this case is a combination of the Hausdorff distance which in general is a measure of the difference between two sets *X* and *Y*:

(13) $$d_{H(X,Y)}=\max\{\underset{X}{\sup}\underset{y\in Y}{\inf}d(x,y),\underset{y\in Y}{\sup}\underset{x\in X}{\inf}d(x,y)\}$$

where *d*(*x*, *y*) is the (metric) distance between the points *x* and *y*. This definition directly provides a goodness of fit between the perimeter of a spore and an egg shape.

Given a closed curve defining a set *S* (for example an egg shaped object) and a blob *B*. Consider both sets to be simply connected subsets of the plane ${\mathbb{R}}^{2}$. The following curve integral defines a distance between these sets.

(14) $$d_{L^{2}}=\sqrt{\int_{S}|d_{\partial B}(r){|}^{2}\mathrm{ds}}$$

where *d*_∂*B*_(***r***) is the distance between ***r*** and the perimeter ∂*B*. Fig. 9 shows an example where a procedure fits an egg-shaped form to the perimeter of a spore where the goodness of fit is a linear combination between the Hausdorff and the *L*^2^ distance (Eqs. (13), (14)). This figure also shows the axis along the spore (cf Section “Axis of blobs” above).

The outer curve around the spore of Fig. 9 provides position for estimates of “normals” to the irregular border of the blob inside it. A real spore will tend to give gradients mainly in the direction of these normals. This feature can contribute within a check if the spore is real or not. The outer ring also provides a check for correlation between the colour inside the blob (covering a potential spore) and colours outside. The colour of the spore tends not to have correlations with its background. A strong correlation between the colour inside the blob and its surroundings, indicates the blob does not reflect a spore.

**Fig. 10:**
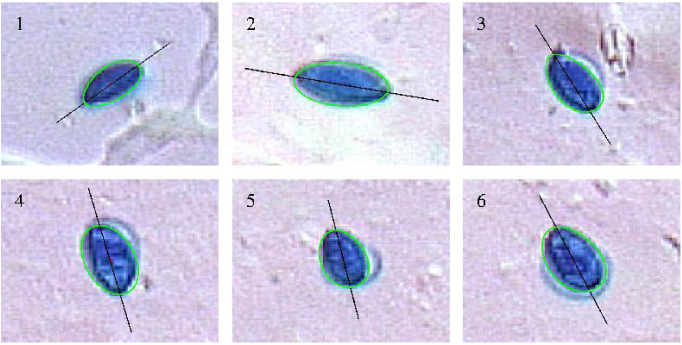
Examples of spores and their egg shape fit (numbers refer to Fig. 2).

Fig. 10 shows egg form projections of spores in Fig. 2. The egg shaped are apparently restricted. The main three-dimensional axis of the spores may tilt relatively to the microscope slide plane. This will affect the observed forms on the image. Hence the egg shapes can be round.

## Discussion and conclusion

The main intention of the above proposal for computerised treatment of microscope slides, is to save cost of labour for current activity without large investments with uncertain utility. Assume only a small fraction of many images show spores to be counted. The present image analysis can in this case serve as a tool to sort out this subset of images for further analysis. Even for example 50 percent over-classification will in this case still save time of labour. A web-based visually based control and correction of classification further saves labour and efforts to count spores.

The present way of classification depends on parameters for segmentation of images in addition to three restricted parameters for egg forms (cf Eqs. (10), (11)) and fitting criteria (Eq. (13) or (14)). It therefore facilitates cost-sensitive computerised learning to optimise classification with respect to the cost of labour and cost of final miss-classification.

Bonner et al. [3] proposed production of imagery data ready for common particle counting. The present proposal is potentially more flexible and can be adapted to data and purpose after data preparation. Benyon et al. [2] attempted to apply large numbers of features for classification. Optimisation of classification in this case requires reduction of the set of features.

Precise figures for miss-classification cannot here be meaningfully provided since it highly depends on concentration of spores and their background in images. Spores of interest often tend to arrive at traps in bursts in the way that there is typically long periods without spores and short periods when many spores arrive. The likelihood for a dust particle in an image to be classified as a spore, depends on the ratio between concentrations of spores and dust particles there.

Actual spinoffs from computerised microscopy are image archiving, accumulation and transfer of knowledge on data treatment and estimation of parameters not otherwise available. Manual microscopy is normally restricted to take place in a laboratory and performed by an available specialist. These restrictions are less an issue for a computerised approach.

Elements of the computerised approach may provide opportunities for remotely controlled real time monitoring of the pathogenic fungus. Also note that the capacity of automatic identification of spores may also be relevant for search in image archives.

## Footnotes

1 http://www.ada2012.org/.

2 http://www.mathematische-basteleien.de/eggcurves.htm.
